# Radiologically Determined Sarcopenia Predicts Morbidity and Mortality Following Abdominal Surgery: A Systematic Review and Meta-Analysis

**DOI:** 10.1007/s00268-017-3999-2

**Published:** 2017-04-06

**Authors:** Keaton Jones, Alex Gordon-Weeks, Claire Coleman, Michael Silva

**Affiliations:** 1 Nuffield Department of Surgical Sciences, University of Oxford, Oxford, UK; 2 Buckinghamshire Healthcare NHS Trust, High Wycombe, Buckinghamshire UK; 3 Department of Hepatobiliary and Pancreatic Surgery, Oxford University Hospitals NHS Foundation Trust, Oxford, UK; 4 CRUK Centre for Radiation Oncology, Radiobiology Research Institute, Department of Oncology, University of Oxford, Churchill Hospital, Roosevelt Drive, Oxford, OX3 7LE UK

## Abstract

**Background:**

Individualised risk prediction is crucial if targeted pre-operative risk reduction strategies are to be deployed effectively. Radiologically determined sarcopenia has been shown to predict outcomes across a range of intra-abdominal pathologies. Access to pre-operative cross-sectional imaging has resulted in a number of studies investigating the predictive value of radiologically assessed sarcopenia over recent years. This systematic review and meta-analysis aimed to determine whether radiologically determined sarcopenia predicts post-operative morbidity and mortality following abdominal surgery.

**Method:**

CENTRAL, EMBASE and MEDLINE databases were searched using terms to capture the concept of radiologically assessed sarcopenia used to predict post-operative complications in abdominal surgery. Outcomes included 30 day post-operative morbidity and mortality, 1-, 3- and 5-year overall and disease-free survival and length of stay. Data were extracted and meta-analysed using either random or fixed effects model (Revman^**®**^ 5.3).

**Results:**

A total of 24 studies involving 5267 patients were included in the review. The presence of sarcopenia was associated with a significant increase in major post-operative complications (RR 1.61 95% CI 1.24–4.15 *p* = <0.00001) and 30-day mortality (RR 2.06 95% CI 1.02–4.17 *p* = 0.04). In addition, sarcopenia predicted 1-, 3- and 5-year survival (RR 1.61 95% CI 1.36–1.91 *p* = <0.0001, RR 1.45 95% CI 1.33–1.58 *p* = <0.0001, RR 1.25 95% CI 1.11–1.42 *p* = 0.0003, respectively) and 1- and 3-year disease-free survival (RR 1.30 95% CI 1.12–1.52 *p* = 0.0008).

**Conclusion:**

Peri-operative cross-sectional imaging may be utilised in order to predict those at risk of complications following abdominal surgery. These findings should be interpreted in the context of retrospectively collected data and no universal sarcopenic threshold. Targeted prehabilitation strategies aiming to reverse sarcopenia may benefit patients undergoing abdominal surgery.

**Electronic supplementary material:**

The online version of this article (doi:10.1007/s00268-017-3999-2) contains supplementary material, which is available to authorized users.

## Introduction

Despite significant improvements in surgical outcomes over recent decades, morbidity and survival following major abdominal surgery still poses challenges. In order to deploy targeted pre-operative risk reduction strategies, risk prediction needs to be accurate on an individual level. The current risk prediction methods include the American Society of Anaesthesiologists (ASA) classification [1], physiological and operative severity score for the enumeration of mortality and morbidity (PPOSSUM) [2] and cardiopulmonary exercise testing (CPET) [3]. These methods amongst others either fail to account for the functional status of patients, or require additional pre-operative hospitals visits which may be costly, time-consuming and unavailable at certain sites. In addition, targeted strategies may be deployed during the pre-operative period with the aim of reversing sarcopenia. Sarcopenia, initially used to describe the loss of lean muscle mass associated with ageing, is now well a documented feature of systemic conditions including inflammatory states, cancer, cachexia, chronic malnutrition and in response to chemotherapy [4]. Sarcopenia leads to reduced mobilisation, suboptimal deep breathing and inability to perform simple activities of daily living [5, 6], partly explaining the increased post-operative morbidity and mortality observed in these patients.

Cross-sectional imaging is routinely performed pre-operatively for the staging of cancer and pre-operative planning. Cross-sectional views of trunk musculature provide an easily obtained objective method for estimating lean muscle mass [7].

Availability of peri-operative cross-sectional imaging has led to an increase in the number of observational studies assessing the relationship between sarcopenia and surgical outcomes. A number of studies have reported significantly worse post-operative morbidity and mortality, as well as reduced long-term survival, in patients with radiological evidence of sarcopenia [8–13]. Whilst recent systematic reviews have described the results of studies focusing on specific gastrointestinal malignancies [14–16], to our knowledge this is the first review and meta-analysis examining the predictive value of radiologically assessed lean muscle mass in patients undergoing any abdominal surgery.

## Method

### Search strategy

The Meta-analysis of Observational Studies in Epidemiology (MOOSE) and Preferred Reporting Items for Systematic Review and Meta-analyses (PRISMA) were consulted throughout this review. A qualified medical librarian conducted the literature search. The following databases were searched for relevant studies: CENTRAL (via Cochrane Library September 2015), EMBASE (via OVID 1974 to September 2015) and MEDLINE (via PubMed 1946 to September 2015). The search strategy used text words and relevant indexing to capture the concept of radiologically assessed sarcopenia used to predict post-operative complications in abdominal surgery. The full search strategy can be viewed in the supplementary material. The following trial registers were searched: ClinicalTrials.gov (www.clinicaltrials.gov September 2015), WHO International Clinical Trials Registry Platform (www.who.int/ictrp September 2015) and UK Clinical Research Network Study Portfolio (http://public.ukcrn.org.uk/search September/2015). A total of 6526 records were retrieved after removal of duplicate manuscripts. Searches did not exclude studies based on publication status or language. Reference lists of key articles and the grey literature were hand-searched.

### Inclusion criteria

Inclusion criteria were established prior to the literature search. Studies reporting the prevalence of sarcopenia and outcomes in adult patients (>18 years) following abdominal surgery were sought. At least one of the following outcomes was required: post-operative mortality (30 days following surgery), post-operative complications, Clavien–Dindo complications, critical care dependency, length of stay, disease-free survival (recurrence of the primary tumour or metastases in cancer patients), overall survival and graft loss (transplantation).

Abdominal surgery was defined as surgery involving the abdominal cavity, including patients undergoing gastrointestinal, hepatobiliary, pancreatic, endocrine, urological, gynaecological and transplantation surgery for both elective and emergency indications. Assessment of lean muscle mass was limited to studies reporting radiological assessment methods, including computed tomography, magnetic resonance and dual-energy X-ray absorptiometry.

### Exclusion criteria

Patients undergoing abdominal interventions other than surgery, including percutaneous radiological procedures were excluded. Studies reporting lean muscle mass as a continuous measure or failing to define a sarcopenic population were also excluded. Subcutaneous surgery not breaching the peritoneum, including abdominoplasty, and patients undergoing oesophagectomy via a thoracic approach were also excluded.

### Study selection

Following removal of duplicates, two investigators screened abstracts independently, and those meeting the inclusion criteria were selected for full-text review.

### Data extraction

Data were extracted independently by two investigators and discrepancies resolved following further review of the full article. Extracted data included age, sex distribution, ethnic characteristics, malignant status, site of primary pathology, grade and stage of tumour, exposure to chemotherapy or radiotherapy, imaging modality and image analysis technique, sex-specific muscle measures, body mass index (BMI), length of stay, complications (any complication and Clavien–Dindo grades), mortality, disease-free survival and overall survival. Authors were contacted in order to obtain raw data where summary data or odds or hazard ratios were reported, or if any further data clarification was required.

### Quality assessment

Investigators independently reviewed each full-text article, assessing quality using the Newcastle–Ottawa assessment scale for each of the outcome measures.

### Risk of bias assessment

The Cochrane collaboration risk of bias assessment tool (chapter 8.5a) was used with additional domains relevant to this review. Domains included image capture, training of assessor, inter-observer reliability, selection bias, allocation concealment, blinding of assessors, incomplete outcome data and selective reporting. Each domain was allocated either a low, unclear or high-risk score and summary data presented using the traffic light system. Funnel plots for each meta-analysis were visually inspected and interpreted in the context of the individual comparisons (Supplementary Fig. 1).

### Heterogeneity assessment

Heterogeneity was estimated using Cochran’s Q statistic, and the percentage of variation in meta-analysed outcomes that could be attributed to sources other than sampling error (*I* ^2^) also was calculated. An *I* ^2^ > 50% was considered to represent a chance of substantial heterogeneity, and >75% considerable heterogeneity.

### Sensitivity analysis

Where the weighting of individual studies within meta-analyses was deemed to be significant (>25%), sequential removal and analysis was performed. Significant results were those resulting in a *p* value that was no longer significant. If there were significant heterogeneity in terms of study population, additional sensitivity analyses were performed as described above.

### Statistical analysis

Freeman–Tukey arcsine transformation was applied for analyses where abstracted proportions had values of zero or one [17]. Heterogeneity amongst study estimates was quantified using the *I* ^2^ and associated test for heterogeneity. Where significant heterogeneity (>75%) was apparent, the DerSimonian and Laird random effects [18] method was used to pool estimates, with inverse-variance weights. Otherwise, the Mantel–Haenszel fixed effect (FE) method was applied [19].

## Results

### Study characteristics

**Fig. 1:**
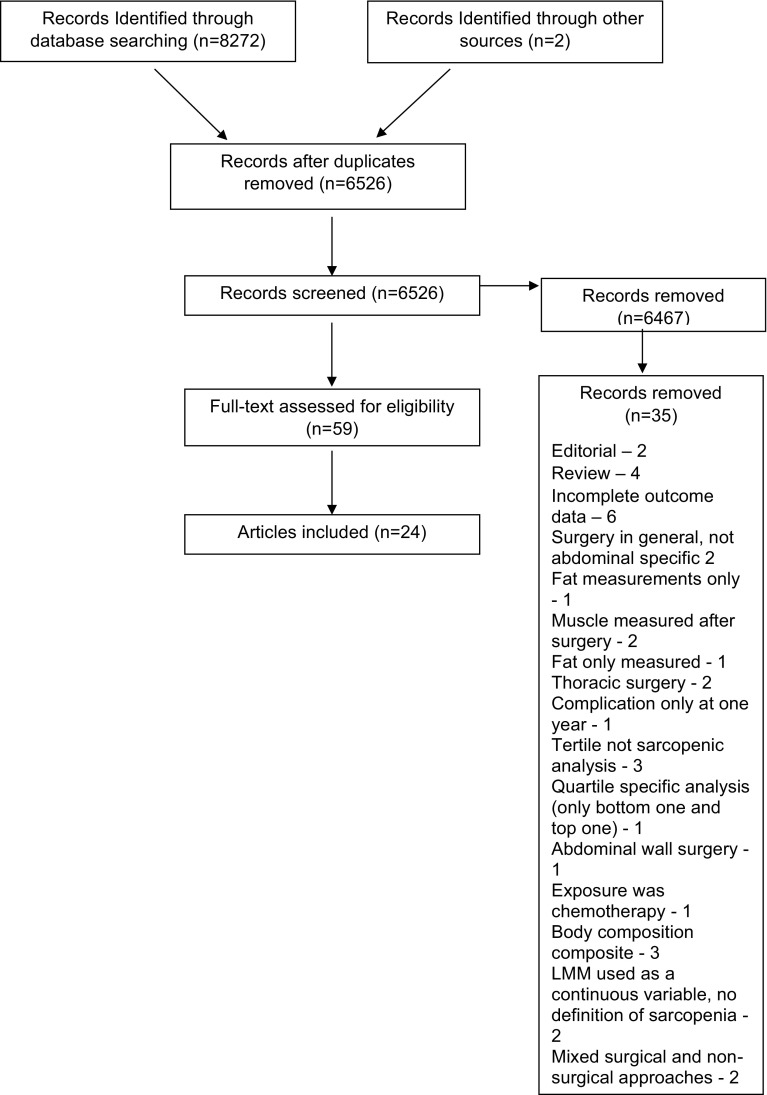
PRISMA flow diagram showing identification of studies and reasons for exclusion

A total of 8272 records were identified from database searching, of which 24 were included in the review [8–10, 20–38]. Nine studies involved patients undergoing hepatobiliary surgery, 4 pancreatic surgery, 4 colorectal surgery, 3 urological surgery, 2 oesophago-gastric surgery and 2 transplant surgery (liver). Five authors were contacted via email on at least two separate occasions to request clarification or to provide further data, three authors responded with raw data which was included in the meta-analysis. The PRISMA flow diagram can be seen in Fig. 1, which includes the reasons for removal of studies.

### Quality, bias and heterogeneity assessment

All of the studies were cohort studies, and quality was high (median 8, range 5–9, see Table 1). A number of parameters in the risk of bias assessment were not reported and therefore scored as unclear. Where bias was assessed, it was generally determined to be low risk, with selection bias being the most frequently reported high-risk domain (Table 2). Following inspection of funnel plots for all analyses, asymmetry was apparent for total complications. An absence of studies in the bottom left side of the plot suggests the possibility of reporting bias, where small studies demonstrating no risk reduction in non-sarcopenic patients are not published. This asymmetry may also be explained by true heterogeneity as those studies towards the right-hand side of the plots represent hepatobiliary cohorts where the greatest relative risk increase was detected.

**Table 1:** Characteristics of included studies

Author	Year	Journal	Country	Study period	No. of patients	Primary pathology	Malignancy	NOS	Modality	Vertebral level	Area assessed	Software used	HU	Sarcopenic	No sarcopenia
Amini	2015	J Gastroint Surg	USA	1996–2014	763	Pancreas	Yes	8	CT	L3	TPV	Ultravisual	Y	192 (25%)	571 (75%)
Coelen	2015	HPB	The Netherlands	1998–2013	100	Bile duct	Yes	8	CT	L3	Psoas	OsiriX	Y	42 (42%)	58 (58%)
Harimoto	2013	British Journal of Surgery	Japan	2004–2009	186	Liver	Yes	8	CT	L3	TMA		Y	75 (40%)	111 (60%)
Higashi	2015	Int J Clin Onc	Japan	2007–2013	144	Liver	Yes	7	CT	L3	Psoas	Synapse vincent	Y	72 (50%)	72 (50%)
Joglekar	2015	J Surg Oncol	USA	2001–2013	118	Pancreas	Yes	7	CT	L3	Psoas		Y	31 (26%)	87 (74%)
Jones	2015	Colorectal Disease	UK	2011–2012	100	Colorectal	Yes	6	CT	L3	Psoas	PACS	N	15 (15%)	85 (85%)
Levolger	2015	J Surg Oncol	The Netherlands	2002–2013	61	Liver	Yes	8	CT	L3	TMA	FatSeg version 4.0	Y	36 (59%)	25 (41%)
Lieffers	2012	British Journal of Cancer	Canada	2002–2006	234	Colorectal	Yes	7	CT	L3	TMA	Slice-o-Matic	N	91 (39%)	143 (61%)
Lodewick	2015	HPB	Holland	2005–2012	171	Liver	Yes	7	CT	L3		Osirix	Y	80 (47%)	91 (53%)
Masuda	2014	Liver Transplantation	Japan	2003–2011	204	Transplant	No	7	CT	L3	Psoas		Y	96 (47%)	108 (53%)
Miyamoto	2015	Annals of Surgical Oncology	Japan	2005–2010	220	Colorectal	Yes	7	CT	L3	TMA	Synapse vincent	Y	55 (25%)	165 (75%)
Montano-Loza	2015	Liver Transplantation	Canada–USA	2000–2012	248	Transplant	No	7	CT	L3	TMA	Slice-o-Matic	Y	112 (45%)	136 (55%)
Okumura	2015	Surgery	Japan	2004–2013	230	Pancreas	Yes	7	CT	L3	Psoas	Aquarius NET server	Y	64 (28%)	166 (72%)
Peng	2011	HPB	USA	2000–2009	259	Liver	Yes	8	CT	L3	Psoas	Ultravisual	Y	41 (16%)	218 (84%)
Peng	2012	Journal of Gastrointestinal Surgery	USA	1996–2010	557	Pancreas	Yes	8	CT	L3	Psoas		Y	139 (25%)	418 (75%)
Peyton	2015	Journal of Endourology	USA	2008–2012	128	Renal	Yes	5	CT	L3	Psoas	Phillips eyesight	Y	32 (33%)	96 (67%)
Psutka	2015	Journal of Urology	USA	2000–2010	387	Renal	Yes	7	CT	L3	TMA	Slice-o-Matic	Y	180 (47%)	207 (53%)
Smith	2014	Journal of Urology	USA	2008–2011	198	Bladder	Yes	8	CT	L3	Psoas	Aquarius iNutrition	Y	77 (39%)	121 (61%)
Tamandl	2015	Eur Radiology	Austria	2006–2013	200	Oesophago-gastric	Yes	7	CT	L3	TMA	OsiriX	Y	130 (65%)	70 (35%)
Tegels	2015	Annals of Surgical Oncology	The Netherlands	2005–2012	152	Oesophago-gastric	Yes	5	CT	L3	TMA	OsiriX	Y	86 (57%)	66 (43%)
van Vledder	2012	British Journal of Surgery	The Netherlands	2001–2009	196	Liver	Yes	8	CT	L3	TMA	MeVisLab	Y	38 (19%)	158 (81%)
van Vugt	2015	Annals of Surgical Oncology	The Netherlands	2005–2013	206	Colorectal	Yes	7	CT	L3	TMA	OsiriX	Y	90 (44%)	116 (66%)
Voron	2015	Annals of Surgical Oncology	France	2006–2012	109	Liver	Yes	8	CT	L3	TMA	Advantage window	Y	59 (54%)	50 (46%)
Valero	2015	J Gastrointest Surg	USA	2000–2013	96	Liver	Yes	8	CT	L3	TPV	ImageJ	Y	47 (49%)	49 (51%)
Total														1820	3447

*NOS* Newcastle–Ottawa scale, *TPV* total psoas volume, *TMA* total muscle area, *TPA* total psoas area, *HU* Hounsfield unit

**Table 2:** Risk of bias summary table. Results for each domain are allocated either low risk (+), high risk (−) or unclear (?)

	Timing of imaging	Trained assessor	Inter-observer reliability	Selection bias	Allocation concealment	Blinding of assessors	Incomplete outcome data	Selective reporting
Amini	+	?	?	+	?	?	+	+
Coelen	+	?	?	+	?	?	+	+
Harimoto	?	?	?	+	?	?	+	+
Higashi	?	?	?	+	?	?	+	+
Joglekar	?	?	?	−	?	?	+	+
Jones	+	?	+	+	?	+	+	+
Levolger	?	+	?	?	?	?	+	+
Lieffers	+	?	?	+	?	?	?	+
Lodewick	?	?	?	−	?	?	+	+
Masuda	+	?	?	+	?	?	+	+
Miyamoto	?	+	?	+	+	+	+	+
Montano-Loza	−	?	?	−	?	?	+	+
Okumura	?	?	?	?	?	?	?	+
Peng 2011	?	?	?	−	?	?	+	+
Peng 2012	?	?	?	−	?	?	+	+
Peyton	?	?	?	?	+	+	+	+
Psutka	?	?	?	−	?	?	+	+
Sabel	?	?	?	?	?	?	+	+
Smith	+	?	?	?	?	?	+	+
Sur	?	?	?	?	?	?	+	+
Tamandl	+	?	?	?	?	?	+	+
Tegels	+	+	?	?	+	+	+	+
Valero	+	+	+	−	?	+	+	+
van Vledder	+	?	?	+	?	?	+	+
van Vugt	?	+	?	?	?	?	+	+
Voron	+	+	?	?	+	?	+	+

### Muscle quantification techniques

All of the included studies used computed tomography to quantify muscle mass, with no alternative quantification methods used in studies excluded following full-text review. In addition, all included studies used the third lumbar vertebra as the landmark for muscle measurement. Alternative landmarks used in other studies include L4 [39], the umbilicus [40] and iliac crests [41]. A majority of studies used image quantification software to calculate surface area, manually drawing around the border of the muscle, or alternatively measuring the antero-posterior and transverse diameter [10]. Quantification by either a trained assessor or radiologist was reported in eight studies [10, 23, 33, 34, 37, 38, 42, 43]. Most used either total lumbar muscle area (TLA) or total psoas area (TPA) with one study using total psoas volume [34] and one using both TPA and TPV [8]. All area measurements were corrected for patient height (Table 1). Sarcopenia was defined as lean muscle mass below a specific threshold based on either previously published parameters or internally derived based upon sensitivity analyses. Thirteen (54%) of the included studies used previously published thresholds to define sarcopenia [9, 10, 12, 13, 21, 28, 32, 37, 38, 44–46], whilst the remaining 11 (44%) defined sarcopenia internally [8, 22, 26, 29–31, 34, 36, 43, 47, 48]. Sex-specific thresholds were frequently calculated in-house for each study based on the local population, with values for TPA ranging from 391 to 414 mm^2^/m^2^ in women, and 468 to 562 mm^2^/m^2^ in men. For TLA, values ranged from 370 to 414 mm^2^/m^2^ in women, and 437 to 550 mm^2^/m^2^ in men. The median values were 475 mm^2^/m^2^ for men and 386 mm^2^/m^2^ for women. The largest thresholds were reported in Western study populations [13], with the difference between Western and Asian populations prompting Higashi et al. [21] to use different thresholds within their study cohort.

### Patient demographics and clinicopathological data

A total of 5267 patients were included across the 24 studies. Thirty-five percentage (1820) patients were sarcopenic, with rates varying from 15% (colorectal [10]) to 66% (liver transplantation [28]). Median age was 65 years, and 60% of patients were male. Ninety-three percentage (4876) patients were operated on for malignancy, with the remaining patients undergoing liver transplantation for both malignant and benign indications [26, 28]. Of the patients who were staged using the TNM classification, 12% (359) were stage 1, 40% (1137) stage 2, 38% (1078) stage 3 and 10% (298) stage 4. Thirty-three percentage (613) had well-differentiated tumours, 56% (1037) moderately differentiated tumours and 11% (217) poorly differentiated tumours.

### Complications

**Fig. 2:**
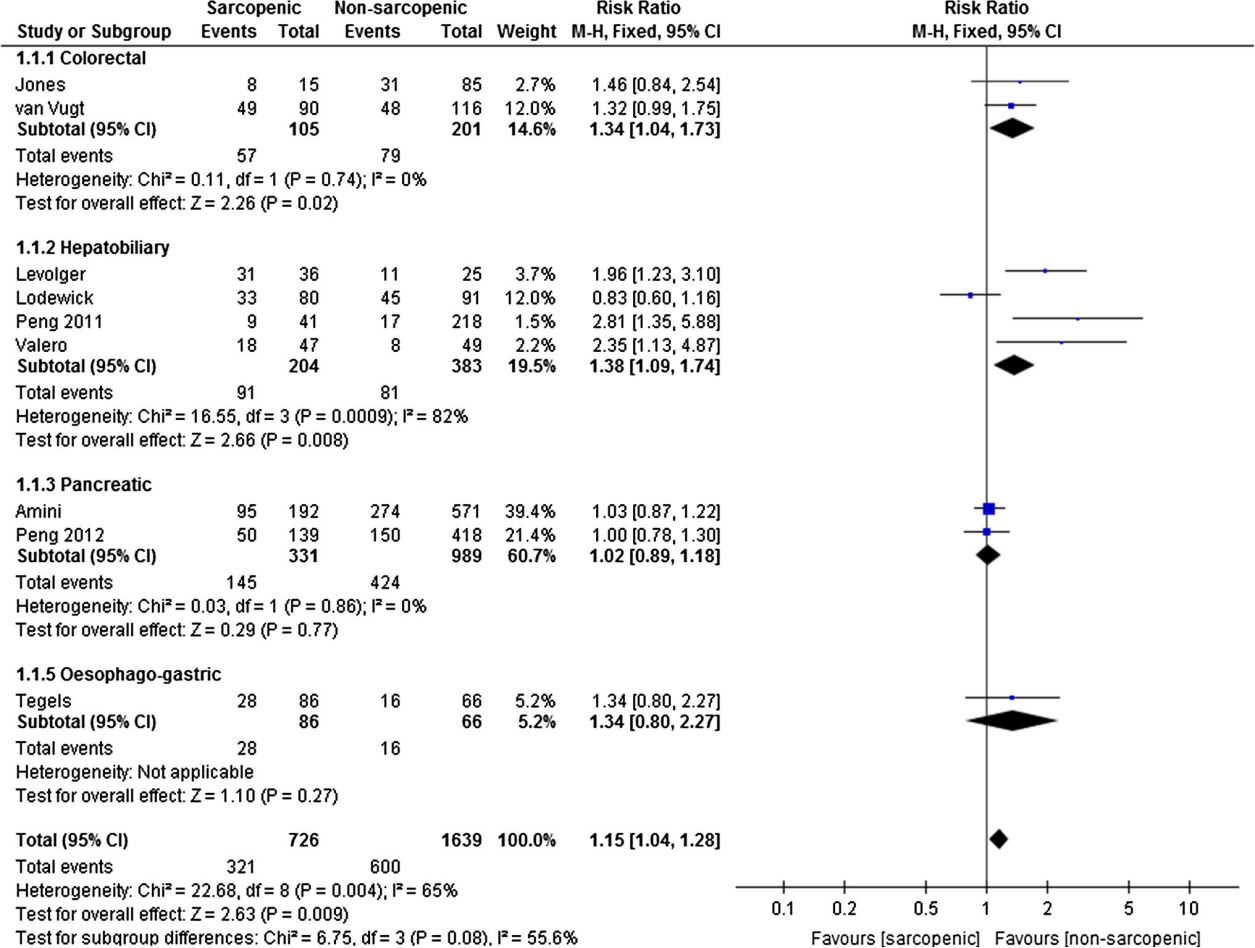
Forest plots comparing overall (any) complications in sarcopenic versus non-sarcopenic patients. A Mantel–Haenszel fixed effects model was used to meta-analyse the data

**Fig. 3:**
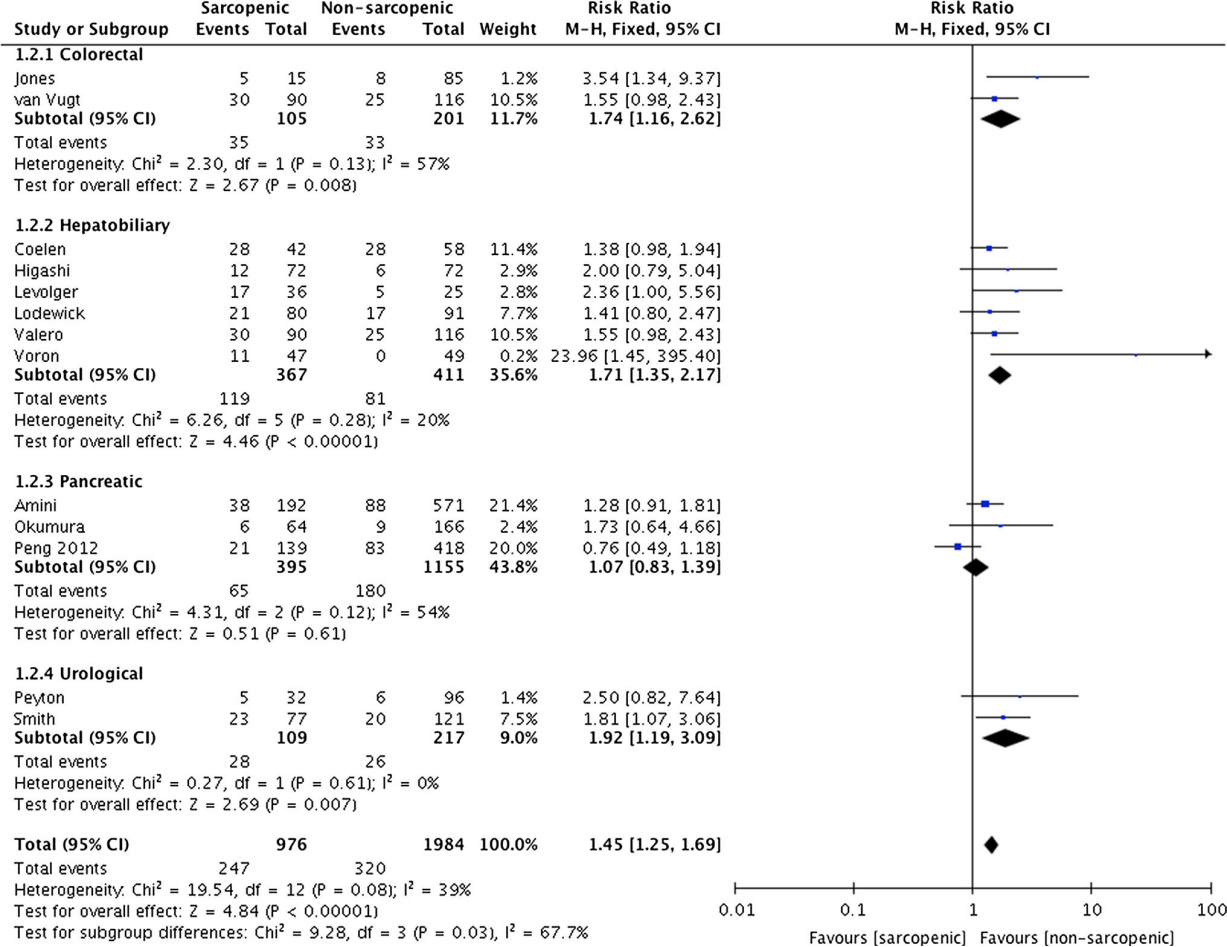
Forest plots comparing major (Clavien–Dindo > 3) complications in sarcopenic versus non-sarcopenic patients. A Mantel–Haenszel fixed effects model was used to meta-analyse the data. Subgroup analysis has been performed for colorectal (1.2.1), hepatobiliary (1.2.2), pancreatic (1.2.3) and urological surgery (1.2.4)

Total complications included all Clavien–Dindo graded complications as well as all other study specific complications. There was a 15% increased risk of any complication, with 54% of sarcopenic patients suffering complications compared with 37% of non-sarcopenic patients (RR 1.15 95% CI 1.04–1.28 *p* = 0.009), though there was significant heterogeneity amongst studies (*I* ^2^ 67%, see Fig. 2). The greatest effect size was seen in colorectal and hepatobiliary surgery (RR 1.75 95% CI 1.06–2.87 *p* = 0.03, OR 1.72 95% CI 1.15–2.58 *p* = 0.008, respectively). Sarcopenia increased the risk of major complications (Clavien–Dindo > grade 3) by 61%, with 25% of sarcopenic patients suffering major complications compared with 16% of non-sarcopenic patients (95% CI 1.24–4.15 *p* = <0.00001 *I* ^2^ 43%, see Fig. 3). Following subgroup analysis, although there was a 7% increased risk of major complications in patients undergoing pancreatic surgery, which failed to reach significance (95% CI 0.83–1.39 *p* = 0.61, see Fig. 3). The baseline characteristics, including tumour stage and grade, were comparable across the three studies involving pancreatic surgery [8, 30, 49]. Of note, the study period overlaps for the cohorts included in the studies by Peng et al. [30] and Amini et al. [8], attributed to the same institution. The more recent report by Amini et al. [8] used total psoas volume to quantify muscle mass, compared with psoas cross-sectional area which was used by the previous study by Peng et al. [8, 30]. Whilst both methods determined 25% of patients to be sarcopenic, the volumetric technique predicted both overall and major complications. Exclusion of the earlier paper by Peng et al. from the subgroup analysis reduced heterogeneity to 0% and increased the relative risk from 9 to 33%, though this failed to reach statistical significance (*p* = 0.09).

### Early post-operative mortality

**Fig. 4:**
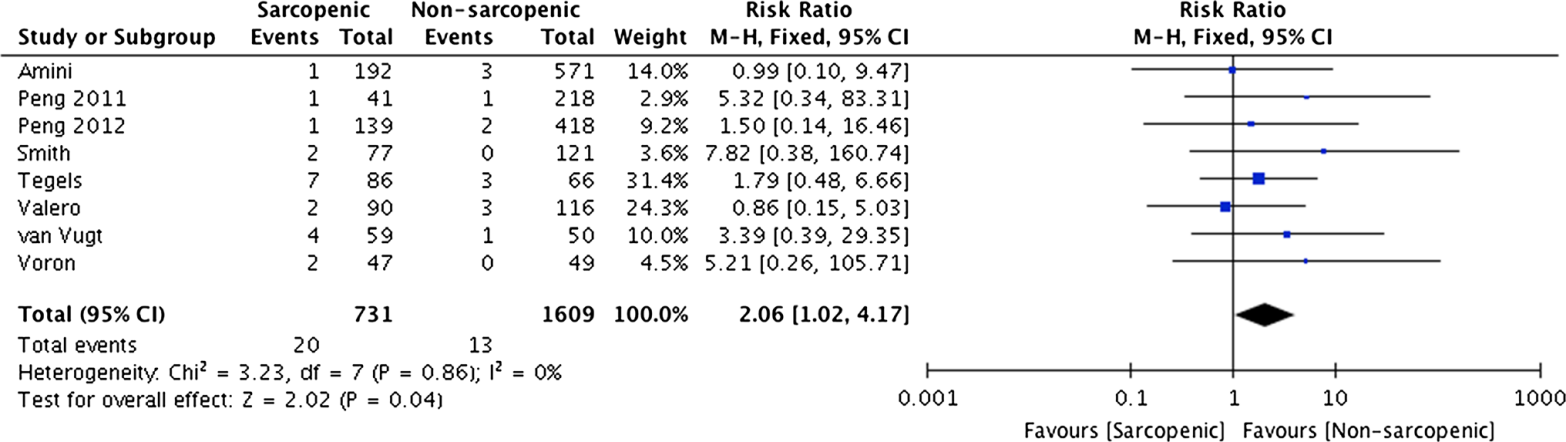
Forest plot comparing 30-day mortality in sarcopenic versus non-sarcopenic patients. A Mantel–Haenszel fixed effects method was used to meta-analyse the data

The presence of sarcopenia significantly increased the risk of post-operative mortality with 2.7% of sarcopenic patients dying within 30 days compared with 0.8% in the non-sarcopenic group (RR 2.06 95% CI 1.02–4.17 *p* = 0.04, see Fig. 4). Sarcopenia was also associated with an increased risk of 90-day mortality (10% sarcopenic vs. 2.5% non-sarcopenic, RR 3.66 95% CI 2.10–6.38 *p* = <0.0001, Supplementary Fig. 2) with no heterogeneity in both analyses (*I* ^2^ 0%, see Fig. 4).

### 1-, 3- and 5-year mortality

**Fig. 5:**
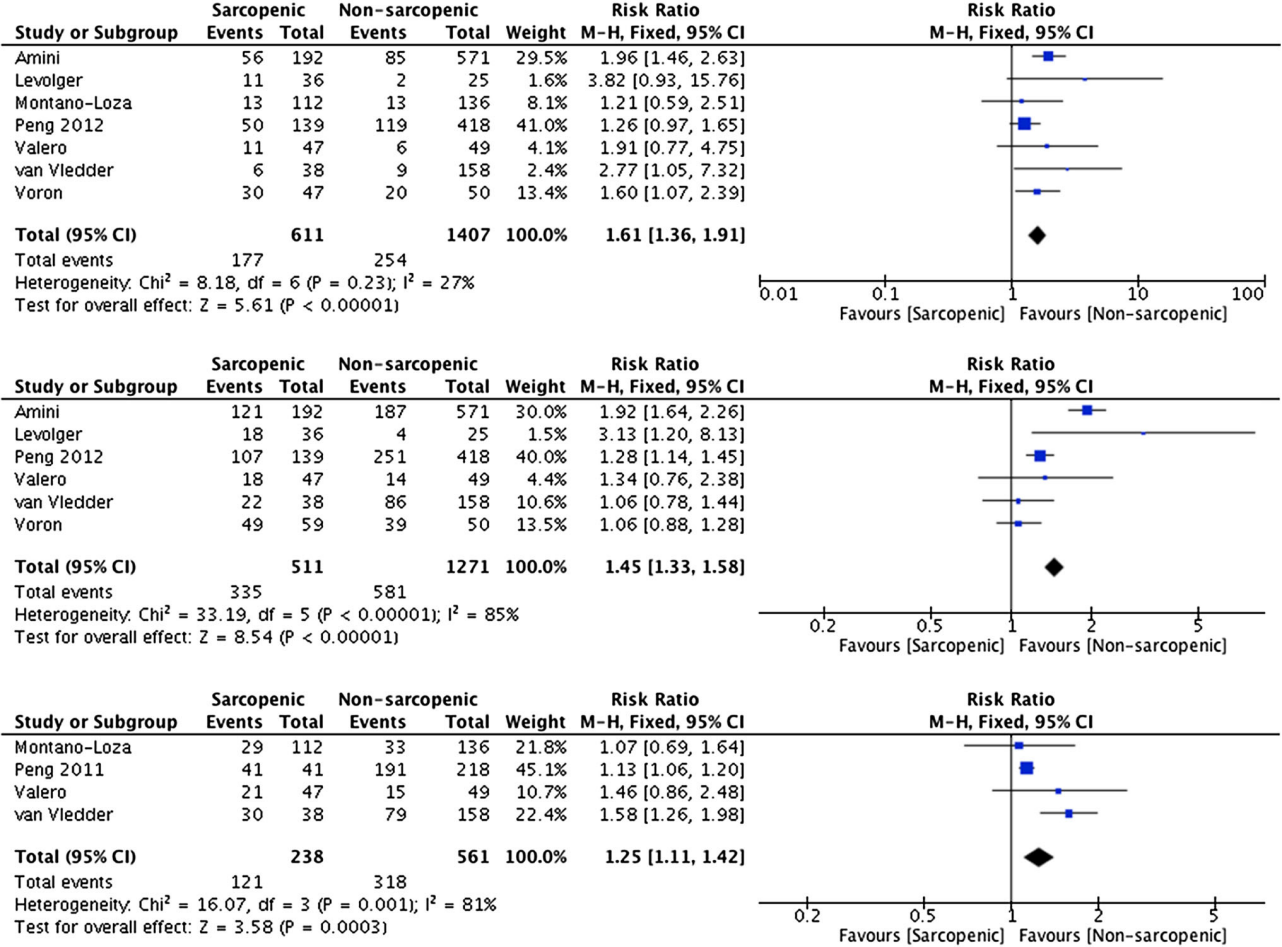
Forest plots comparing overall survival in sarcopenic versus non-sarcopenic patients. The first plot presents 1-year survival, the second 3-year survival and the third 5-year survival. A Mantel–Haenszel fixed effects method was used to meta-analyse the data

Sarcopenia predicted 1-, 3- and 5-year survival, with the risk decreasing from 61% for 1-year, 45% for 3-year and 25% for 5-year (29% sarcopenic vs. 18% non-sarcopenic RR 1.61 95% CI 1.36–1.91 *p* = <0.0001 (1-year), 66% sarcopenic vs. 45% non-sarcopenic RR 1.45 95% CI 1.33–1.58 *p* = <0.0001 (3-year), 50% sarcopenic vs. 56% non-sarcopenic RR 1.25 95% CI 1.11–1.42 *p* = 0.0003 (5-year), see Fig. 5). Studies reporting 1-, 3- and 5-year survival included liver (1019), pancreas (1022) and transplant (169) patients. In addition, median overall survival was shorter in patients with sarcopenia in 8 from a total of 10 studies [11, 20, 31, 34, 37, 43, 47, 50], ranging from 17.7 to 69.7 months in sarcopenic patients, to 18–146 months in non-sarcopenic patients (Supplementary Fig. 3). In the 2 studies where median survival in sarcopenic patients exceeded non-sarcopenic patients, the difference was non-significant [8, 45].

### Disease-free survival

**Fig. 6:**
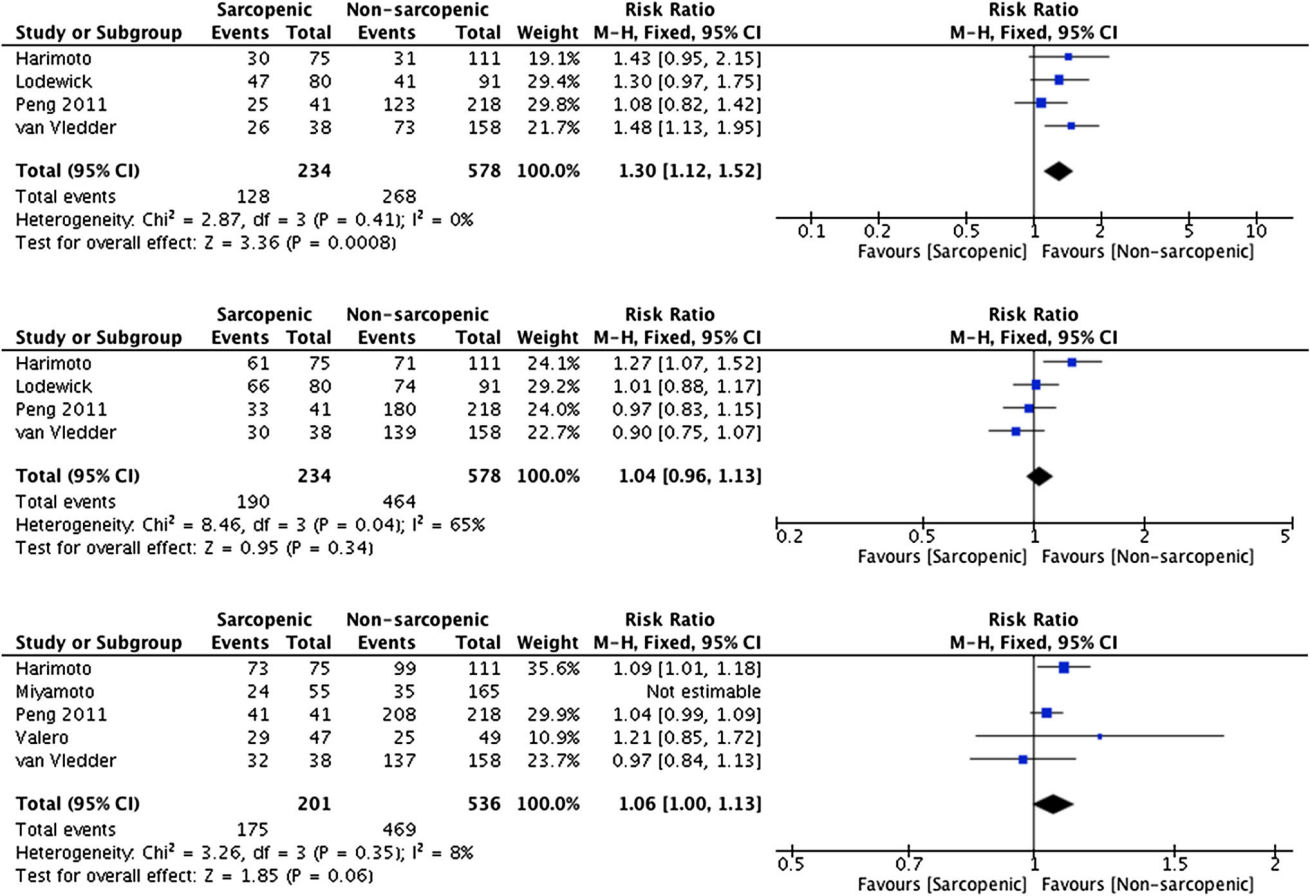
Forest plots comparing disease-free survival in sarcopenic versus non-sarcopenic patients. The first plot presents 1-year survival, the second 3-year survival and the third 5-year survival. A Mantel–Haenszel fixed effects method was used to meta-analyse the data

All five studies reporting disease-free survival included patients with malignant disease. One-year disease-free survival was significantly worse in patients with sarcopenia, with 55% of sarcopenic patients suffering recurrence compared with 45% of non-sarcopenic patients (RR 1.30 95% CI 1.12–1.52 *p* = 0.0008, see Fig. 6). Whilst the risk was increased for 3-year disease-free survival (81% sarcopenic vs. 80% non-sarcopenic, RR 1.04 95% CI 0.96–1.13 *p* = 0.04, see Fig. 6), the relative risk increase was significantly reduced at 4%, and for 5-year disease-free survival, there was no difference between sarcopenic and non-sarcopenic groups (RR 1.06 95% CI 1.00–1.13 *p* = 0.06, see Fig. 6).

### Length of stay

Ten studies including 2766 patients reported data pertaining to length of hospital stay. Length of stay was increased in patients with sarcopenia in 8 studies, with 6 identifying a significant difference (see supplementary Fig. 4). The remaining two studies showed no significant difference in length of stay between sarcopenic and non-sarcopenic patients [30, 35]. There were also comparable overall complications [30] and major complications [35] between sarcopenic and non-sarcopenic patients in these studies including oesophago-gastric and pancreatic cancer patients. In addition, three of the studies collected data on intensive care unit (ICU) stay, reporting significantly increased ICU stay in patients with sarcopenia [24, 29, 51, 52].

## Discussion

This meta-analysis of 24 studies including 5267 patients aimed to determine the predictive value of radiologically determined sarcopenia for outcomes following abdominal surgery. Lean muscle mass was quantified using peri-operative cross-sectional imaging and the presence of sarcopenia predicted post-operative complications, mortality, 1-, 2- and 5-year overall survival and 1- and 3-year disease-free survival. In addition, sarcopenia was frequently associated with increased length of stay and intensive care dependency. The inclusion of patients undergoing any abdominal surgery in this study facilitated meta-analytical evaluation of the data, the first reported to our knowledge.

Nearly all (93%) patients included in the analysis underwent oncological resection surgery, the remainder being transplant recipients with significant comorbidities [11, 53]. The paucity of evidence available for non-oncological surgery is likely due to the incidence of post-operative morbidity following major resectional surgery, where risk prediction may result in the greatest overall benefit to patients. There are limited data available on radiologically quantified lean muscle mass in the normal population, which is reflected in the lack of robust criteria for defining sarcopenia based on these measurements. Investigators have responded by determining their own thresholds based upon local populations and sensitivity analyses. Joglekar et al. suggested the introduction of a universal cut-off value for defining sarcopenia. However, given the heterogeneity in prevalence amongst different populations, it may be more appropriate to continue calculating the thresholds based on local demographics.

Independent muscle quantification fails to take into account the corresponding functional status of patients. Although the relationship is assumed, none of the included studies performed an assessment of muscle function. The correlation is clearer when muscle loss results from ageing [54], but is likely to be part of more complex interactions in cancer patients where cachexia and chemotherapeutic agents may affect function as well as volume [55]. In an attempt to improve predictive sensitivity, some investigators have sought to take advantage of 3D imaging and software programmes by calculating total muscle volume and adjusting for fatty infiltration and the presence of blood vessels using Hounsfield Units, with encouraging results [8, 34]. Accounting for fatty infiltration may go some way in mitigating the inability of volume alone to predict function. In a weighted risk stratification score developed by Wagner et al. [56], Hounsfield unit average calculation alone improved sensitivity in an elderly population undergoing hepatobiliary surgery. In studies using total psoas volume (TPV), the measurement technique involved measuring the cross-sectional area at sequential slices to a total of 55cms psoas length. This was then corrected for patient height and resulted in a sarcopenia prevalence of 48.9% and 19.9%, respectively, compared with 45.8 and 25.1 as measured by total psoas area (TPA) alone in the same patients [8, 34]. In both of these studies, TPV was better at predicting outcomes following surgery and remained so following multivariate analysis. We were unable to identify studies specifically reporting the number of patients determined sarcopenic by one method but not the other. The emerging data, however, would suggest that volumetric measurements combined with density analyses might be more sensitive.

Sarcopenia was a more significant predictor of major complications (61%) compared with overall complications (15%). In terms of respiratory and thromboembolic complications, this may be as a result of reduced deep breathing and mobilisation. In cases of sepsis, anastomotic leaks and other major complications that are less likely to be associated with functional status, this may reflect the underlying functional and nutritional status of the patient. We were unable to find any reports examining the link between loss of muscle and the status of the primary lesion on a biological level. However, an association between primary tumour biology and the extent of sarcopenia is supported by the reduction in effect size over time in terms of both overall and disease-free survival. Whilst sarcopenia predicted 1-, 3 and 5-year mortality, the risk reduced over time (61, 34 and 25%, respectively). A similar pattern was observed for disease-free survival. This supports the suggestion that any observed loss in muscle mass is likely as a result of a combination of factors associated with the presence of malignancy. There are insufficient data from patients with benign disease along with a lack of any follow-up muscle measurements to fully support this theory.

Following subgroup analysis, sarcopenia failed to significantly predict overall and major complications in patients undergoing pancreatic surgery. Study by Peng et al. was the only study in the pancreatic subgroup failing to show increased morbidity in the sarcopenic group [30]. The authors themselves are unable to explain this finding, but suggest it may be due to the inherently low complication rate in their high-volume centre. Interestingly, a more contemporary report from the same institution using total psoas volume to determine lean muscle mass found that sarcopenia was associated with both overall and major complications. When the previous study using cross-sectional area measurement is removed from the subgroup, significance is still not reached (*p* = 0.09). This finding is somewhat difficult to explain, especially in light of emerging evidence suggesting that sarcopenia is strongly associated with the development of post-operative pancreatic fistula formation, a leading cause of morbidity in patients undergoing pancreatic resection [57, 58].

On inspection of the funnel plot for total complications, there is a suggestion of asymmetry towards greater significance. There is an absence of smaller studies reporting less significant effects. Whilst this may be explained by publication bias, it may also be related to the outcome measure being used to report the outcomes (Clavien–Dindo). Clavien–Dindo accounts for complications ranging from minor (*I*) through to death (*V*). Studies using the classification for outcome coding may be expecting a higher incidence of serious complications, whilst studies with few serious complications may not benefit from the uniform coding of minor complications.

The abundance of emerging contemporary evidence investigating the prognostic role of sarcopenia has resulted in a number of recent review articles [14, 15, 59, 60]. With focus on a specific abdominal malignancy in two such reviews, including colorectal [61] and hepatobiliary surgery [15], meta-analysis was precluded due to data heterogeneity between the included studies. A recent review by Shachar et al. examined the prognostic value of sarcopenia in patients with solid organs tumours, irrespective of disease site. This allowed for meta-analysis and included 7843 patients in total. A majority of the study population comprised non-surgical patients with advanced or metastatic disease. Interestingly, the relative risk increase for overall survival in sarcopenic patients was not significantly different between non-metastatic, metastatic or mixed cohorts. These findings, along with our results, suggest that the aetiology of sarcopenia in cancer populations is multifactorial and tumour burden may be one of many contributing factors. Of note, sarcopenia failed to predict overall survival in the pancreaticobiliary subgroup. Whilst the patients are different from the surgical population, with metastatic or advanced disease, these results are in keeping with our findings. Although this analysis provides useful insight into the prognostic value of sarcopenia in cancer patients, it cannot address the question of whether sarcopenia predicts post-operative complications and mortality.

Sarcopenia as a concept in surgical risk prediction is attractive due its potential reversibility. In elective surgical patients, there is a small window of opportunity from the point at which scans are acquired to the date of surgery. The approach during this period should be multi-faceted and targeted to the individual patient. A combination of strategies targeting inflammation, reduced exercise capacity, secondary anorexia and reduced food intake have been suggested in the context of cancer-related cachexia [62, 63]. In the targeting of muscle mass and strength, the largest body of evidence relates to ageing and muscle loss. A number of studies have reported modest increases in mass and capacity following relatively short anaerobic resistance training [64–67]. Whilst reduced length of stay and critical care dependency has been observed in cardiothoracic patients receiving prehabilitation programmes, there are limited data examining the subsequent impact on post-operative outcomes following abdominal surgery [68–70]. In addition, cost-effectiveness and patient satisfaction needs to be accounted for if pre-operative interventions are to be considered by budget commissioners. In order to properly address the question of whether prehabilitation in sarcopenic patients can reduce complications and mortality following surgery, clinical trials need to be considered.

Limitations of this study include the retrospective observational nature of the included articles. In addition, based on funnel plot analysis, the results may be affected by publication bias.

The majority of included studies used muscle volume thresholds to define sarcopenia based on individual study populations. Whilst this accounts for inherent geographical differences, the limited generalisability means those wishing to further investigate sarcopenia may need to perform internal sensitivity analyses in order to determine thresholds.

## Conclusion

In conclusion, these data support a role for radiologically determined sarcopenia in post-operative prognostication. The retrospective nature of included studies, along with the lack of consensus surrounding sarcopenic thresholds, limits the generalisability of the results. Nonetheless, there may be a potential role for targeted pre-operative interventions in sarcopenic patients aimed at improving post-operative outcomes. Future work needs to determine the efficacy of interventions targeting muscle mass and function in the pre-operative setting. In addition, the benefit of sarcopenia reversal in surgical patients would need to be investigated using prospective trial designs.

## Electronic supplementary material

Below is the link to the electronic supplementary material.

Supplementary material 1 (DOCX 438 kb)
