# Supplementary material for: Radiologically Determined Sarcopenia Predicts Morbidity and Mortality Following Abdominal Surgery: A Systematic Review and Meta-Analysis
Source: World J Surg. 2017 Apr 6;41(9):2266–79. doi: 10.1007/s00268-017-3999-2 (PMC5544798; doi:10.1007/s00268-017-3999-2)
Supplement: Supplementary file 1 — Supplementary material 1 (DOCX 438 kb) [file 268_2017_3999_MOESM1_ESM.docx]

**Supplementary Figure 1** – Funnel plots for each meta-analysis carried out. A - All complications, B – Major complications, C – 30-day mortality, D – 90-day mortality, E – 1-year mortality, F – 3-year mortality, G – 5-year mortality, H – 1-year disease-free survival, I – 3-year disease-free survival, J – 5-year disease-free survival.


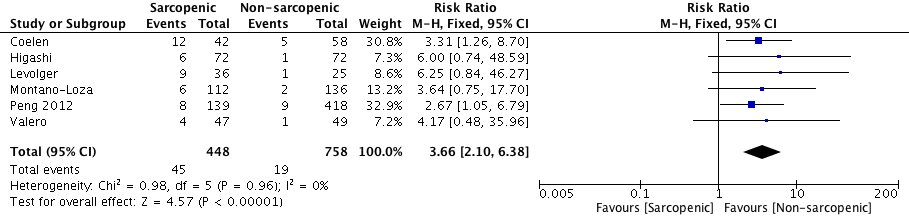


**Supplementary Figure 2** - Forest plot comparing 90-day mortality in sarcopenic versus non-sarcopenic patients. A Mantel-Haenszel fixed effects method was used to meta-analyse the data.

**Supplementary Figure 3** – Summary data graph showing median overall survival (months) in sarcopenic versus non-sarcopenic patients.

**Supplementary Figure 4** – Summary data graph showing mean length of stay (days) in sarcopenic versus non-sarcopenic patients.
